# Multi-platform metabolomics analyses of a broad collection of fragrant and non-fragrant rice varieties reveals the high complexity of grain quality characteristics

**DOI:** 10.1007/s11306-015-0925-1

**Published:** 2016-01-23

**Authors:** R. Mumm, J. A. Hageman, M. N. Calingacion, R. C. H. de Vos, H. H. Jonker, A. Erban, J. Kopka, T. H. Hansen, K. H. Laursen, J. K. Schjoerring, J. L. Ward, M. H. Beale, S. Jongee, A. Rauf, F. Habibi, S. D. Indrasari, S. Sakhan, A. Ramli, M. Romero, R. F. Reinke, K. Ohtsubo, C. Boualaphanh, M. A. Fitzgerald, R. D. Hall

**Affiliations:** Plant Research International, Wageningen University and Research Centre, Droevendaalsesteeg 1, Wageningen, The Netherlands; Grain Quality, and Nutrition Centre, International Rice Research Institute, DAPO 7777, Metro Manila, Philippines; Centre for BioSystems Genomics, P.O. Box 98, 6700 AB Wageningen, The Netherlands; Biometris-Applied Statistics, Wageningen University and Research Centre, Droevendaalsesteeg 1, Wageningen, The Netherlands; Laboratory of Plant Physiology, Wageningen University and Research Centre, Droevendaalsesteeg 1, Wageningen, The Netherlands; Netherlands Metabolomics Centre, Einsteinweg 55, 2333 CC Leiden, The Netherlands; Max-Planck-Institute of Molecular Plant Physiology (MPIMP), Am Mühlenberg 1, 14476 Potsdam-Golm, Germany; Plant and Soil Science Section, Department of Plant and Environmental Sciences, Faculty of Science, University of Copenhagen (UC), Thorvaldsensvej 40, 1871 Frederiksberg C Copenhagen, Denmark; The National Centre for Plant and Microbial Metabolomics, Rothamsted Research, West Common, Harpenden, Herts AL52JQ UK; Ubonratchathani Rice Research Centre, Ubon Ratchathani, Thailand; Rice Programme, National Agricultural Research Centre, Islamabad, Pakistan; Grain Quality Division, Rice Research Institute of Iran (RRII), Km 5 Tehran Rd, 41996-13475 Rasht, Islamic Republic of Iran; Indonesian Center for Rice Research (ICRR) BB Padi, Jl. Raya 9, Sukamandi, Subang, 41256 Jawa Barat Indonesia; Cambodian Agricultural Research and Development Institute, CARDI Rd, Phnom Penh, Cambodia; Pusat Penyelidikan Padi dan Tanaman Industri, MARDI, Seberang Perai Beg Berkunci 203 Pejabat Pos Kepala Batas, 13200 Seberang Perai Pulau, Penang Malaysia; Rice Chemistry and Food Science Division, Philippine Rice Research Institute, Maligaya, Science City of Muñoz, 3119 Nueva Ecija Philippines; Graham Centre for Agricultural Innovation, Agricultural Institute (An Alliance Between NSW Department of Primary Industries and Charles Sturt University), Wagga Wagga, NSW Australia; Department of Applied Biological Chemistry, Faculty of Agriculture, Niigata University, Niigata, Japan; Department of Plant Science and Agricultural Resources, Faculty of Agriculture, Khon Kaen University, Khon Kaen, 40002 Thailand; Rice and Cash Crops Research Centre, National Agriculture and Forestry Research Institute, PDR, Vientiane, Lao; School of Agriculture and Food Science, University of Queensland, St Lucia, QLD 4072 Australia; Plant Breeding, Genetics and Biotechnology Division, International Rice Research Institute, DAPO 7777, Metro Manila, Philippines

**Keywords:** Rice, Multi-platform analysis, Jasmine, Basmati, Grain quality, PLS-DA

## Abstract

The quality of rice in terms not only of its nutritional value but also in terms of its aroma and flavour is becoming increasingly important in modern rice breeding where global targets are focused on both yield stability *and* grain quality. In the present paper we have exploited advanced, multi-platform metabolomics approaches to determine the biochemical differences in 31 rice varieties from a diverse range of genetic backgrounds and origin. All were grown under the specific local conditions for which they have been bred and all aspects of varietal identification and sample purity have been guaranteed by local experts from each country. Metabolomics analyses using 6 platforms have revealed the extent of biochemical differences (and similarities) between the chosen rice genotypes. Comparison of fragrant rice varieties showed a difference in the metabolic profiles of jasmine and basmati varieties. However with no consistent separation of the germplasm class. Storage of grains had a significant effect on the metabolome of both basmati and jasmine rice varieties but changes were different for the two rice types. This shows how metabolic changes may help prove a causal relationship with developing good quality in basmati rice or incurring quality loss in jasmine rice in aged grains. Such metabolomics approaches are leading to hypotheses on the potential links between grain quality attributes, biochemical composition and genotype in the context of breeding for improvement. With this knowledge we shall establish a stronger, evidence-based foundation upon which to build targeted strategies to support breeders in their quest for improved rice varieties.

**Electronic supplementary material:**

The online version of this article (doi:10.1007/s11306-015-0925-1) contains supplementary material, which is available to authorized users.

## Introduction

Rice is not only the most important global food crop, it is also a major export commodity and source of income for many developed and developing countries (Khush and Juliano 1985). In particular, fragrant rice types such as basmati, produced in India and Pakistan, and jasmine which is widely cultivated in S.E. Asia, are specifically favoured not only locally, but also outside their production regions where they find major and increasing markets, particularly in Europe, N. America and Australia. For example, the volume of Thai rice imported into the EU increased more than three fold in the period 2000–2006 (Supakornchuwong and Suwannaporn 2012) and in Australia, imports from Thailand are required to meet a very large shortfall in fragrant rice each year. The global market for basmati export was worth $US1 billion in 2007 (Archak et al. 2007) and has since continued to rise. These two types of fragrant rice command the highest prices on the international market, being up to three times more expensive than high quality, non-fragrant rice (www.oryza.com).

The basmati rices of the highest quality are single line selections from traditional varieties of the Punjab region, shared by India and Pakistan, and are in the *aromatic* germplasm class (McNally et al. 2009). The high quality jasmine varieties, such as Khao Dawk Mali (KDML) 105 from Thailand, are also single line selections, but from traditional *indica* varieties and were domesticated in the Greater Mekong Sub region (GMS) (McNally et al. 2009). Aroma originated in the *aromatic* germplasm class (Kovach et al. 2009) and introgression into *indica* varieties occurred probably as a result of hybridisation during domestication. Traditional varieties are generally low yielding, and many countries, including Thailand, Pakistan and India, invest heavily in programs attempting to combine the quality of these aromatic rices with superior agronomic adaptation (Fitzgerald et al. 2009). Fragrant rice is also found in the other germplasm classes, but many of these are improved varieties which tend to be consumed predominantly in the countries where they have been developed and cultivated.

A major limitation for breeding programs aiming to increase the yield potential of fragrant rice is a lack of understanding of the particular fragrance that is characteristic of basmati and jasmine rices. All fragrant rice varieties contain the fragrant compound 2-acetyl-1-pyrroline (2AP) (Buttery et al. 1982), and the genetics driving 2AP accumulation are now well known (Bradbury et al. 2005; Kovach et al. 2009). Currently, 2AP is the only component that rice breeders can select for, either phenotypically or genetically (Bryant and McClung 2011). However, it has already been recognised that the importance of 2AP to rice fragrance has been over-emphasised relative to the many other (unknown) contributory compounds (Limpawattana et al. 2008). More than 100 other volatile compounds have been found in rice grains (Buttery et al. 1983, 1988), each of which has the potential to influence the sensory experience. Even compounds present at very low levels can still impart significant fragrant/flavour notes when they have low odour thresholds, as is the case for 2AP (Champagne 2008). Rice fragrance and flavour are highly complex sensory attributes, involving a wide range of chemical compound classes. Our previous work has shown that the grains from aromatic rices from Southeast Asia, South Asia and Central Asia differ significantly both in taste (Champagne et al. 2010), and in the metabolomic composition of the grains (Calingacion et al. 2014).

Metabolomics is an analytical technology focused on profiling myriad of small metabolites present in biological samples (Hall 2006; Saito et al. 2006). As an untargeted approach, it has the potential to play a key role in defining better what is meant by grain quality, and the technology can help identify those components which either positively or negatively influence sensory characteristics without any great requirement for prior knowledge (Stewart et al. 2011). A range of approaches has been developed, generally centred around combinations of separation (e.g. gas chromatography; liquid chromatography; capillary electrophoresis) and detection (e.g. mass spectrometry; nuclear magnetic resonance) techniques (Hall and Hardy 2012; Hall 2011). Following on from some of the more traditional approaches, metabolomics is now also becoming established as a valuable technology to evaluate rice quality (Calingacion et al. 2012; Fitzgerald et al. 2009).

Uniquely, in the study described here, we have deliberately sourced a wide range of fragrant and non-fragrant rice varieties specifically from their countries of origin through the International Network for Quality Rice (INQR). These materials have been produced by INQR members under the cultivation conditions for which each specific variety has been bred. We have therefore been able to avoid the potential limitations of growing all varieties at a single location which would have entailed that most would have been cultivated under unadapted conditions. Furthermore, in contrast to many previous reports, usually based on store-bought materials, we can guarantee that pure samples of confirmed varietal identification have been used in the present study. We have also had full knowledge and control of the post-harvest handling of the grains (storage, transport and polishing) which can also greatly affect final sensory quality. Within the EU consortium META-PHOR (www.meta-phor.eu) we have had access to six contrasting but complementary analytical platforms capable of profiling a broad range of small molecules and mineral micronutrients (Hall 2007). These platforms have already demonstrated their value by providing new insights into both the primary and secondary metabolite profiles of rice breeding materials (Calingacion et al. 2012) and have the potential to identify biochemical markers appropriate for use in breeding programmes for different types of fragrant rice (Calingacion et al. 2014).

The objectives of this study were to investigate: (i) differences in the metabolomic signature of fragrant rice from each germplasm class, (ii) the effect of storage on both jasmine and basmati rices, and (iii) differences between improved and traditional varieties of both basmati and jasmine rices. The results clearly indicate both the complexity and the dynamism of rice metabolomic profiles and indeed, emphasise how much we still do not know about which metabolites are involved in c.q. causal of, key quality traits.

## Materials and methods

### Plant materials

Thirty one rice (*Oryza sativa* L.) varieties were produced in their local environments and resulted in a total of 44 samples (Table S1). Samples included fragrant varieties from the *aromatic*, *sadri*, *indica*, *tropical japonica*, and *temperate japonica* germplasm classes (Garris et al. 2005; McNally et al. 2009), as well as non-fragrant varieties from each germplasm class. Basmati varieties from the Punjab, and basmati-like samples from Central Asia, are from the *aromatic* and *sadri* classes respectively. The others were jasmine style rices from the *indica* and the *japonica* classes. The set included traditional and improved varieties (Table S1). The basmati set included samples that were freshly harvested and also that had been stored for up to 1 year in containers at room temperature, as typically occurs in commercial practice to achieve the quality that basmati consumers prefer. The *sadri* rice from Iran is usually consumed after 3 months storage, and our set included freshly harvested grains as well as grains stored for 3 months at ambient temperature. Fragrant *indica* rice is usually consumed soon after harvest, but samples stored at ambient temperature for 6 months and 1 year were also prepared to compare the aging of the *indica* jasmine with that of the basmati type grains.

Replicate grain samples from each variety were harvested at maturity and sun-dried to an average of 14 % moisture. Freshly harvested or stored paddy from each sample was dehulled (Satake Rice Machine, Tokyo, Japan), and brown grain was polished (Grainman 60-230-60-2AT, Grain Machinery Mfg. Corp., Miami, FL, USA) at IRRI (Philippines). Polished rice samples was couriered from each rice producing country to Plant Research International, Wageningen where they were immediately placed at 4 °C before grinding.

### Metabolomic profiling

Approximately 50 g polished rice of each variety was ground to a fine powder in liquid nitrogen to generate a stock sample which was thereafter stored at −80 °C. Aliquots of these powders were then couriered on dry ice to each metabolomic profiling partner, selected for their expertise with a particular metabolomics platform. Volatile compounds were measured using GC-qMS of the headspace sampled by solid-phase micro extraction (SPME) at PRI (Wageningen UR, NL). Primary polar metabolites were profiled by GC–TOF–MS at MPIMP-Golm (DE), both semi-polar and polar compounds by ^1^H-NMR and direct infusion ESI MS at Rothamsted Research (UK) and, finally multi-elemental analysis was performed using ICP-MS at UC (DK).

#### Headspace analysis of volatile compounds by SPME–GC–MS

Headspace volatiles were collected by solid phase micro-extraction (SPME) using a 65 µm polydimethylsiloxane-divinylbenzene fibre (Supelco, Bellefonte, USA) as described previously (Verhoeven et al. 2012). The volatile compounds were thermally desorbed at 250° C by inserting the fibre for 1 min into the GC injection port (GC 8000, Fisons Instruments, Cheshire, UK). The released compounds were transferred onto the analytical column (HP-5, 30 m × 0.25 mm ID, 1.05 μm film thickness) in splitless mode. The temperature program ran from 45 °C (2-min hold) and rose 5 °C min^−1^ to 250 °C (5-min hold). The column eluent was ionised by electron impact ionisation at 70 eV (MD800 electron impact MS, Fisons Instruments, Cheshire, UK). Mass scanning was done from 35 to 400 m/z with a scan time of 2.8 scans s^−1^. GC–MS raw data were processed using MetAlign software (Lommen 2009) to extract and align the mass signals (s/n ≥ 3). Mass signals below an s/n of 3 were randomized and given a value between 2.4 and 3 times the calculated noise value. Mass signals present in ≤6 samples were discarded. Signal redundancy per metabolite was removed by means of clustering and mass spectra were reconstructed using MSClust (Tikunov et al. 2012). Metabolites were identified by matching the mass spectra of detected metabolites to authentic reference standards and the NIST08, Wiley, and Wageningen Natural Compounds spectral library and by comparison with retention indices in the literature (Strehmel et al. 2008).

#### ^1^H-NMR fingerprinting analysis

The methods previously optimised for ^1^H-NMR profiling of wheat flour (Baker et al. 2006) and ^1^H-NMR-ESI–MS profiling of Arabidopsis (Ward et al. 2010) were followed. To replicate aliquots of rice flour (100 mg) in 1.5 mL Eppendorf tubes was added D_2_O–CD_3_OD (1 mL, 4:1) containing 0.05 % w/v TSP-*d*4 (sodium salt of deuterated trimethylsilylpropionic acid). The contents of the tube were mixed thoroughly and heated at 50 °C in a water bath for 10 min. After centrifugation for 5 min, 800 μL of the supernatant was transferred to a clean Eppendorf tube. To ensure cessation of hydrolytic enzyme activity, the samples were heated at 90 °C in a water bath for 2 min, cooled at 4 °C for 45 min and re-centrifuged for 5 min (still at 4 °C); 700 μL of the supernatant was transferred to a 5-mm NMR tube and a further 50 µL to a glass auto-sampler vial containing 950 µL of H_2_O:methanol (4:1) for DI-MS.

^1^H-NMR spectra were acquired under automation at 300 K on an Avance Spectrometer (Bruker BioSpin, Coventry, UK) operating at 600.0528 MHz and equipped with a 5 mm selective inverse probe. Spectra were collected using the zgpr water suppression pulse sequence with pre-saturation and a 90° pulse and a relaxation delay of 5 s. Each spectrum was acquired using 128 scans of 64 K data points with a spectral width of 7309.99 Hz. Spectra were automatically Fourier transformed using an exponential window with a line broadening value of 0.5 Hz. Phasing and baseline correction were carried out within the instrument software. ^1^H chemical shifts were referenced to -TSP-d_4_ at δ0.00. ^1^H-NMR spectra were automatically reduced, using Amix (Analysis of MIXtures software, *Bruker BioSpin* GmbH, Rheinstetten, Germany), to ASCII files containing integrated regions or ‘buckets’ of equal width (0.01 ppm). Spectral intensities were scaled to the -TSP-d4 region (δ0.05 to −0.05). The ASCII file was imported into Excel for the addition of sampling/treatment details. HCA clustering was carried out in Spotfire (Tibco) using extracted characteristic spectral regions, identified via comparisons to a library of authentic standards, which were reduced to unit variance prior to analysis.

#### Direct infusion ESI–MS fingerprinting analysis (DI-MS)

For direct infusion ESI–MS, 100 µL of each sample was infused into the spectrometer (Esquire 3000, Bruker Daltonics... Bruker Daltonics, Coventry, UK) by flow injection using an Agilent 1100 series HPLC system with degasser, quaternary pump and auto-sampler. The flow rate was 100 µL min^−1^. Mass spectra were recorded from 1.7 to 4.2 min after the sample had entered the flow. Spectra were recorded in both positive and negative electrospray ionization modes on the same sample via an alternating sequence of the two ionisation modes, each time recording an average of 25 scans. The spectra were recorded over an m/z range of 50–1000 using the “smart tuning” function (Bruker Daltonics) with a target mass of m/z 300, a trap-drive and stability of 100 % and a scan speed of 13,000 m/z min^−1^. The spectra were recorded using Ion Charge Control with a maximum accumulation time of 40 ms for 20,000 (negative mode) or 50,000 (positive mode) ions. The nebuliser pressure was 20 psi and the dry gas flow rate was 6 L min^−1^ at 350 °C.

Using Data Analysis 3.2 (Bruker Daltonics) the accumulated positive and negative ion spectra for each sample were separately combined and exported as ASCII files of m/z-intensity pairs. For spectral alignment AMIX software was used to reduce the ASCII files to a single data table for each ionisation mode, containing integrated regions or buckets of equal width (m/z 1). Individual spectral intensities were scaled to total spectrum intensity and exported as a CSV file for annotation in Excel and subsequent PCA analysis in SIMCA-P.

#### Gas chromatography–time of flight mass spectrometry (GC–TOF–MS): derivatized metabolite fractions

Sample extraction, chemical derivatization, injection, instrumentation, chromatogram and data pre-processing were exactly as previously described (Allwood et al. 2009). Retention indices (RI) were generated for each individual chromatogram based on a spiked mixture of alkanes. Data were aligned according to RI and full mass unit into a numerical data matrix using the TagFinder software (Luedemann et al. 2008). Clusters of at least three corresponding mass fragments were selected for relative metabolite quantification. Metabolites were identified by TagFinder supported matching to reference spectra and RI from the Golm Metabolome Database (Luedemann et al. 2008). The matching process was manually supervised with RI deviations <1 % (Strehmel et al. 2008).

### Multi-elemental analysis

Whole grain milled samples were sent to the University of Copenhagen for multi-elemental analysis. The grain samples were surface decontaminated by 3 consecutive rinses in Milli-Q-water (Millipore, Bedford, Massachusetts, USA) and samples were subsequently freeze-dried for 48 h (Christ Alpha 2-4, Martin Christ GmbH, Osterode, Germany). Whole rice grain samples were then digested in 100 mL closed vessels in a microwave oven (Multiwave 3000, Anton Paar, Graz, Austria) for 50 min at 210 °C with a maximum pressure of 40 bar. The digestion medium consisted of 250 mg dry grain sample, 5 mL 65 % ultrapure HNO_3_ (J.T. Baker Instra-Analysed Reagent) and 5 mL 15 % H_2_O_2_ (30 % Extra-Pure, Riedel de Häen, Selze, Germany). After digestion, the samples were diluted to 3.5 % v/v HNO_3_ with ultrapure Milli-Q water (Laursen et al. 2009). Multi-elemental analysis was performed using ICP-MS (Agilent 7500ce, Agilent Technologies, Manchester, UK) tuned in standard mode. The plasma power was operated at 1500 W and the argon carrier and make-up gases were set at 0.82 and 0.17 L min^−1^, respectively. Sample uptake was maintained at approximately 0.6 mL min^−1^ by a PFA micro-flow nebulizer. Elimination of spectral interferences was achieved using an octopole ion guide with the cell gasses helium or hydrogen as described previously (Laursen et al. 2009). Using this instrumental set-up, 13 elements were analysed: Na, Mg, Al, P, S, K, Ca, Mn, Fe, Ni, Cu, Zn and Mo. Seven replicates of a certified reference material NIST 8436 (durum wheat, particle size <200 μm; National Institute of Standards and Technology, Gaithersburgh, MD, USA) were included to validate digestion efficiency and analytical accuracy. Only element concentrations deviating less than ±10 % from the certified mean reference values were accepted. The limit of detection (LOD) was determined as three times the standard deviation of a minimum of 7 blanks. Only data above LOD were included for chemometric analysis.

### Statistical analysis

Raw data obtained by the different platforms were autoscaled (van den Berg et al. 2006). To obtain an overview of the whole data set, principal component analysis (PCA) was performed using the software package SIMCA-P (version 13.0. Umetrics, Umeå, Sweden). The number of significant PC’s was determined using cross validation (Eriksson et al. 2006). Hierarchical cluster analysis, using Euclidean distance and Ward’s linkage method, was applied to depict the similarities of the samples as measured by the different analytical platforms.

To identify the metabolites that are important for describing the differences between the groups, a double cross validated partial least squares discriminant analysis (PLS-DA) was used (Smit et al. 2007; Westerhuis 2008). PLS-DA is a classification technique that creates linear combinations (latent variables) of the original variables to predict the class labels, indicated by zeroes and ones.

A two nested leave-one-out cross validation or double cross validation was used to obtain an unbiased estimate of the prediction error. In this way, class membership of a sample was predicted without its use in the model building process. In the outer cross validation, one sample was set aside, and the model built using the remaining samples. To determine the optimal number of latent variables for this PLS-DA model, the remaining samples were subjected to an inner leave-one-out cross validation. By iteratively repeating this procedure and leaving out all samples one at a time, we obtained a class prediction and model per sample. The number of misclassifications from this procedure was compared to the results from a permutation test (Smit et al. 2007) to check if the results could have been obtained by chance alone.

To find metabolites that are predictive for the different groups, we focused on those that had high absolute values for the regression coefficients in the PLS-DA models. Since the double cross validation procedure yielded as many models as the number of samples that we had, we summarized the many models using rank products (Smit et al. 2007). The absolute regression coefficients from each model were ranked (with rank 1 for the highest absolute value). To summarize the importance of each metabolite, the rank numbers from all models were multiplied together, giving a rank product. Metabolites with a low rank product are typically the most important metabolites, since that metabolite was often at the top of the list of sorted regression coefficients. When rank products are (very) high, the metabolite in question was typically in the lower regions of the sorted list, indicating that often other metabolites were more important.

## Results

Thirty one fragrant and non-fragrant rice varieties from 10 different countries were used in this study. Of these varieties, 23 were fragrant and 8 were not (Table S1). All samples have been analysed by six generally complementary metabolomics platforms: SPME–GC–MS of headspace extracts (518 reconstructed peaks), ^1^H-NMR of 25 % MeOH extracts (95 frequency bins), DI-MS of 25 % MeOH extracts (634 mass signals in negative ionization mode; 367 mass signals in positive ionization mode), GC–TOF–MS of derivatized polar extracts (82 compounds; mainly primary metabolites), and ICP-MS for micronutrients (13 elements). The raw data from these six analytical platforms were concatenated, resulting in a dataset consisting of 1709 variables, and were subjected to different multivariate statistical techniques including principal component analysis (PCA), hierarchical clustering analysis (HCA), and partial least squares-discriminant analysis (PLS-DA).

**Fig. 1:**
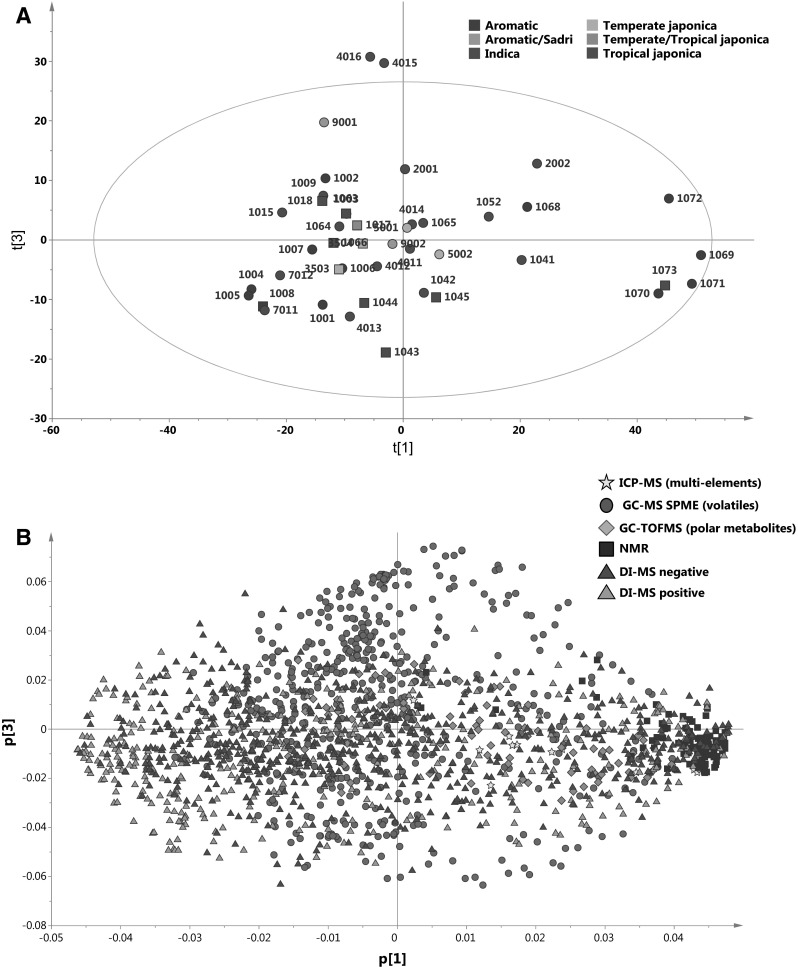
Principal component analysis (PCA) of the metabolome as detected by six different analytical platforms of 23 fragrant and 8 non-fragrant rice varieties. **a** Scores plot showing the first PC and third PC explaining 24.8 and 6.2 % of the variation respectively. *Numbers* next to the *symbols* represent the metaphor code of the rice varieties as given in Table S1. Different colors indicate the different germplasm classes. *Circles* indicate fragrant varieties while *boxes* represent non-fragrant varieties. **b** Loadings plot of the first PC versus the third PC. The five different analytical platforms are indicated by the *different colored symbols* (Color figure online)

Figure 1a represents a PCA of all 31 varieties, combining the data from all six platforms. The first PC, explaining 24.8 % of the total variation, mainly separates the fresh basmati varieties from the majority of the other varieties. Interestingly, IR6 (1073) which is a freshly harvested and non-fragrant variety from the *tropical japonica* germplasm class grown in Pakistan, resembles the non-stored *aromatic* basmati rices from Pakistan with regard to its metabolite profile. In the first two principal components, there are no other notable groupings. The PCA loadings show that the metabolites from the DI-MS and ^1^H-NMR platforms are at the extremes of PC1, whereas metabolites from the other platforms are more randomly distributed (Fig. 1b). Metabolites that were analysed by NMR are highly correlated with and are highly abundant in the fresh basmati samples (Fig. 1a, b). Since the ^1^H-NMR extraction protocol specifically targets polar metabolites such as carbohydrates, amino acids and organic acids the data would suggest that fresh basmati samples are richer in this class of metabolites compared to other varieties. For micronutrients, no systematic differences were found between rice samples across the 13 elements analysed.

**Fig. 2:**
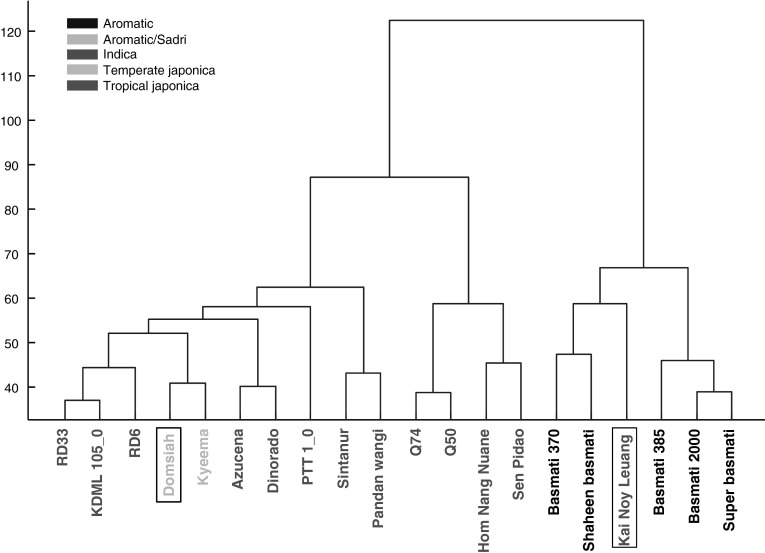
Hierarchical cluster analysis (HCA) of the metabolome as detected by five different metabolomics platforms of 20 fragrant rice varieties that were analyzed soon after harvest (fresh). Samples are colored according to their germplasm class. The boxed label indicates samples which were incorrectly classified by double cross validated PLS-DA (Color figure online)

Figure 2 represents an HCA of all the samples of freshly harvested samples of both jasmine and basmati rice, combined for all platforms. It is clear that the first cluster break separates the jasmine samples from the basmati samples, with two unexpected exceptions: Kai Noy Leuang (KNL) from Lao PDR clusters with the basmati samples, and Domsiah from Iran, clusters with the jasmine samples. There is no consistent pattern of germplasm class in the clustering. Even though major differences in the metabolic profiles of the different varieties can be recognized, the picture is still too complex to answer specific questions. Consequently, to reduce the level of complexity and the number of variables in the data set, a number of logical inter-group comparisons were performed, using the data from individual platforms, to examine in detail the presence of discriminatory compounds between specific sub-groups. We focused on (i) the metabolome of stored basmati varieties from the Punjab and freshly harvested jasmine varieties from Thailand according to the manner in which they are normally consumed; (ii) the metabolic differences between freshly harvested and stored basmati varieties; and (iii) the metabolomic differences between freshly harvested basmati and jasmine varieties. These analyses were also used to reveal which metabolomics platforms are most valuable for discriminatory analyses with the view to delivering selection tools to breeders.

### As rice is marketed: fresh Thai jasmine versus stored basmati varieties

**Fig. 3:**
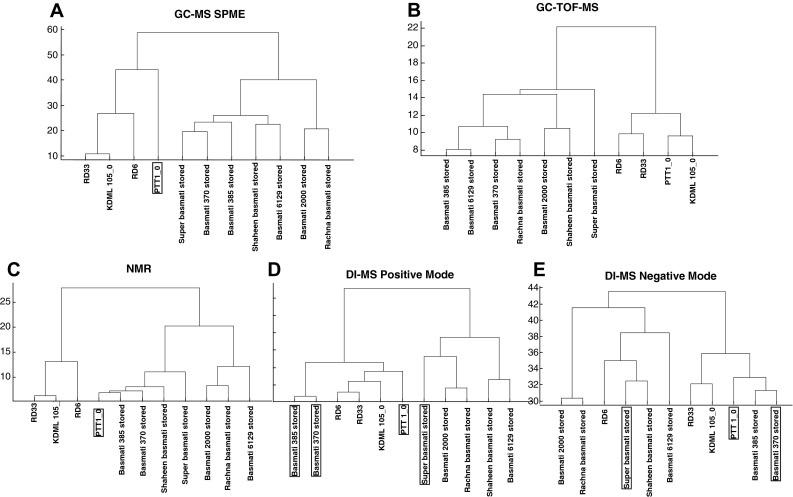
Hierarchical cluster analysis (HCA) of the metabolic profiles of four fresh Thai jasmine varieties and seven stored basmati varieties. Each analytical platform is represented by a separate dendrogram. The *boxed label* indicates samples which were incorrectly classified by double cross-validated PLS-DA

Figure 3 represents dendrograms from an HCA depicting the relationship between metabolite profiles of traditional and improved samples of basmati and jasmine rice, as they would normally be sold and consumed: freshly harvested for jasmine, and stored locally at ambient temperature for about 12 months, for basmati. The volatile and derivatized polar metabolites both showed the expected separation between basmati and jasmine rices (Fig. 3a, b), but the other platforms (Fig. 3c–e) misclassified some of the samples. Statistically, PTT 1 was misclassified by PLS-DA by all platforms except for GC–TOF–MS. However, even though the volatile profile of PTT 1 was significantly different from the Thai varieties, using HCA, PTT 1 still clustered within the sub-group of jasmine varieties (Fig. 3a). Six of the ten most discriminating volatile compounds are more abundant in the stored basmati samples than in the jasmine varieties, but none of these could be unambiguously annotated (Table 1). The most discriminating polar (GC–TOF–MS) compounds included the organic acids tetradecanoic acid, nonanoic acid and fumaric acid, which were more abundant in basmati varieties, while erythronic acid and its lactone derivative, raffinose, and 2-amino-malonic acid were more abundant in jasmine varieties. For metabolites detectable by NMR, PTT 1 appears to be more similar to the basmati rices according to HCA and was consequently misclassified by PLS-DA. Both positive and negative DI–MS techniques were unable to show the expected classification. Only when these two DI–MS modes were used to look at semi-polar metabolites were the improved basmati varieties observed to have a different profile compared to the traditional varieties (Basmati 370 and 385) (Fig. 3d, e).

**Table 1:** List of metabolites detected by the analytical platforms that significantly contribute to the metabolic differences of fresh and stored basmati varieties as determined by partial least squares-discriminant analysis (PLS-DA) (Color table online)

Metabolites were ranked in descendant order of the rank products that was determined by double cross validation. Mean and standard deviation (sd) of the metabolites of the different compared varieties is given. For clarity, colour codes indicate the higher (red) and lower (blue) values for each

### Fresh versus stored basmati varieties

**Fig. 4:**
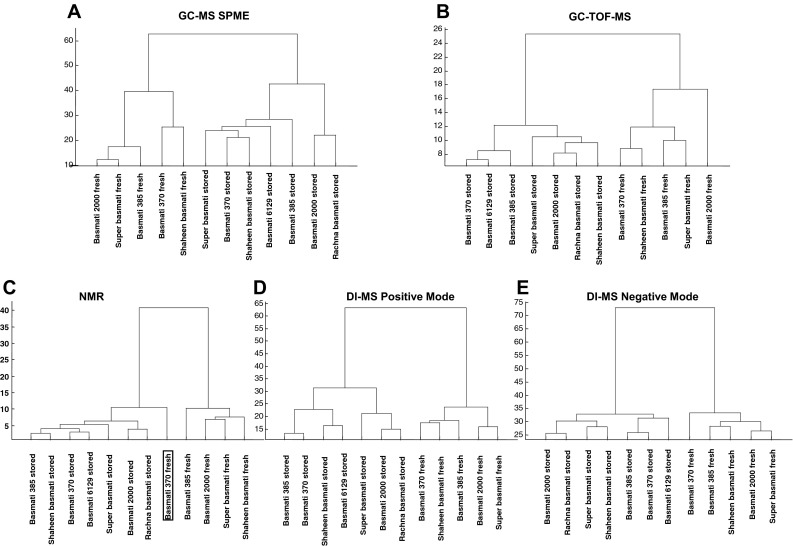
Hierarchical cluster analysis (HCA) of the metabolic profiles of fresh and stored basmati varieties. Each metabolomics platform is represented by a separate dendrogram. The *boxed label* indicates the sample was incorrectly classified by double cross-validated PLS-DA

Freshly harvested basmati could be discriminated from stored basmati by every analytical platform using HCA (Fig. 4). With the exception of the ^1^-NMR profile of Basmati 370, all samples were also correctly classified by PLS-DA (Fig. 4). The metabolites most responsible for the separations are shown in Table 2. Of the 10 most discriminatory volatile compounds, three were more abundant in fresh basmati while seven had higher values in stored basmati varieties (Table 2). The differences between stored and fresh basmati varieties with regard to polar metabolites, as analysed by GC–TOF–MS and ^1^H-NMR, were mainly based on metabolites more abundant in the fresh basmati samples. The differences detected by DI-MS positive mode were less pronounced than for the other platforms. Very few of the 50 most discriminating metabolites can yet be annotated (Table 2).

**Table 2:** List of metabolites detected by the analytical platforms that significantly contribute to the metabolic differences of fresh traditional Thai jasmine varieties and stored basmati varieties as determined by PLS-DA (Color table online)

Metabolites were ranked in descendant order of the rank products that was determined by double cross validation. Mean and standard deviation of the metabolites of the different compared varieties is given. For clarity, colour codes indicate the higher (red) and lower (blue) values for each

### Fresh Thai jasmine versus fresh basmati varieties

**Fig. 5:**
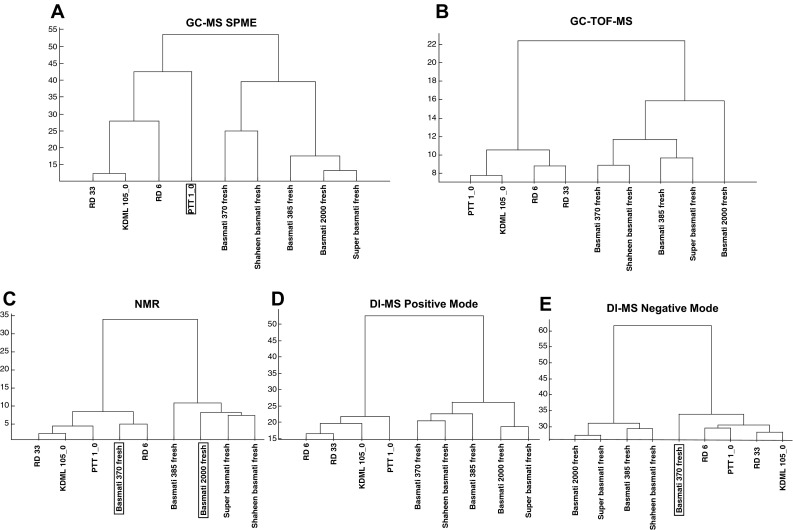
Hierarchical cluster analysis (HCA) of the metabolic profiles of fresh Thai jasmine varieties and fresh basmati varieties. Each metabolomics platform is represented by a separate dendrogram. The *boxed label* indicates samples that were incorrectly classified by double cross-validated PLS-DA

To determine whether the metabolites in basmati and jasmine differ immediately after harvesting, fresh samples of basmati and jasmine rices were compared (Fig. 5). The overall picture from all platforms clearly indicated that grains of fresh basmati and jasmine differ in their suite of metabolites. The metabolic profile of the improved jasmine variety PTT 1 was significantly different from the other jasmines with regard to the volatile patterns (as determined by PLS-DA), but in spite of this, PTT 1 always still fell in the jasmine sub-cluster (Fig. 5). The profiles of Basmati 370 as analysed by ^1^H-NMR and DI-MS negative mode were not classified as basmati varieties by PLS-DA. Unexpectedly, when analysed by ^1^H-NMR, the improved variety Basmati 2000 clustered using HCA within the basmati samples (Fig. 5c) but was misclassified when using PLS-DA. The metabolites from all platforms that contributed the most to the separation between fresh jasmine and basmati varieties are shown in Table 3.

**Table 3:** List of metabolites detected by the analytical platforms that significantly contribute to the metabolic differences of fresh traditional Thai jasmine varieties and freshly harvested basmati varieties as determined by PLS-DA (Color table online)

Metabolites were ranked in descendant order of the rank products that was determined by double cross validation. Mean and standard deviation of the metabolites of the different of the metabolites is given. For clarity, colour codes indicate the higher (red) and lower (blue) values for each

### Effects of storage on metabolome of Thai jasmine rices

**Fig. 6:**
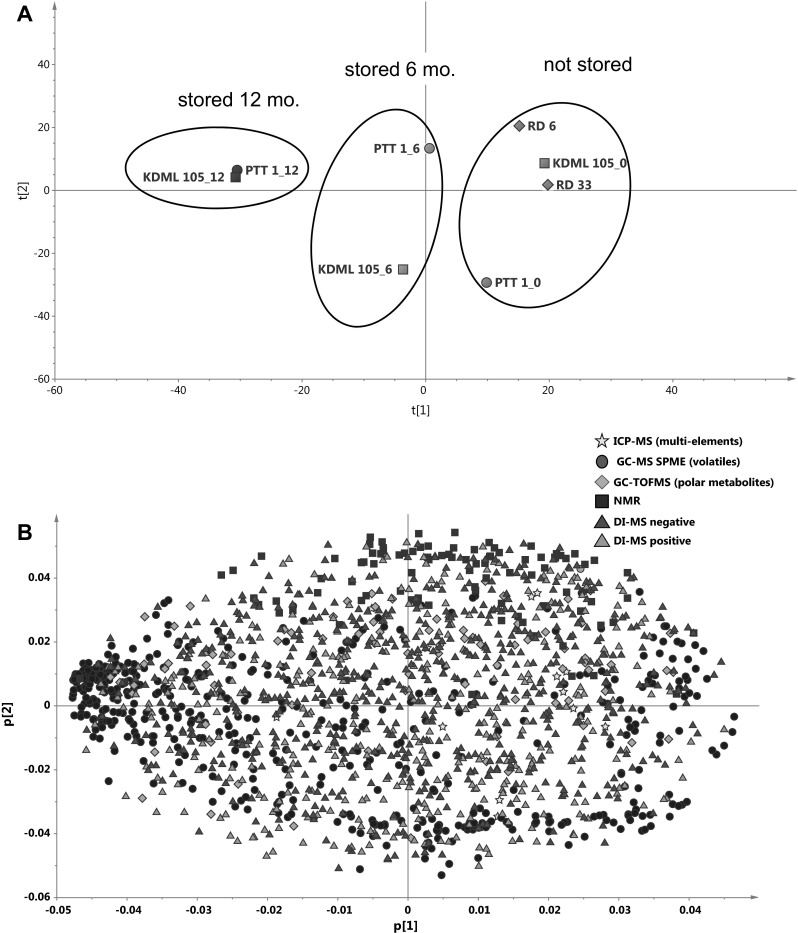

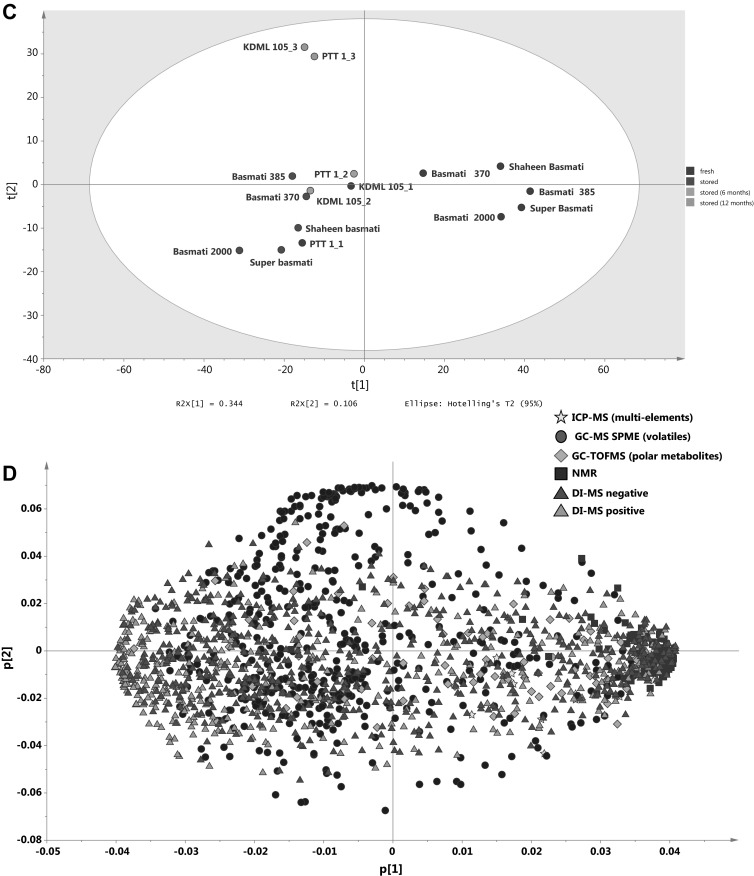
PCA of the metabolome as detected by five different metabolomics platforms of five basmati varieties from the Punjab region and Thai jasmine varieties that were fresh or stored for up to a maximum of 12 months. **a** Score plot of fresh and stored Thai jasmine varieties showing PC1 versus PC2 explaining 24.9 and 18.6 % of the variation respectively. Grain samples were analyzed soon after harvest (*green*), 6 months (*orange*) and 12 months (*red*) after storage at room temperature. **b** Corresponding loading plot of the first PC versus the second PC. **c** Score plot of basmati and Thai jasmine varieties showing PC1 versus PC2 explaining 34.4 and 10.6 % of the variation respectively. **d** Loading plot of the first PC versus the second PC. The five different analytical platforms are indicated by the *different colors* (Color figure online)

While clear changes in the metabolome occur during storage in Basmati rices (Fig. 4) little is known about changes to jasmine rices upon storage. We therefore analysed two important jasmine rices from Thailand: KDML 105 and PTT 1 that were either fresh, stored at ambient temperature for 6 months or for 12 months. In addition, two other fresh fragrant jasmine varieties RD 6 and RD 33 were included as extra reference materials. When combining all metabolic platforms, a clear aging effect could be seen (Fig. 6a). Unsurprisingly, samples that had been stored for 12 months were particularly characterised by an increase/appearance of many specific volatile compounds (Fig. 6b). Interestingly, in the un-stored samples, PTT 1 is separated from the other jasmine rices along PC2, and after 6 months storage both KDML 105 and PTT 1 have moved along PC1, but notably with an opposite separation along PC2. After 12 months of storage, both samples have moved further along PC1, and there is no longer any separation along PC2 (Fig. 6a).

When comparing the profiles of jasmine and basmati rices in response to storage it appears that aging in jasmine rice apparently results in different metabolome changes than for basmati (Fig. 6c). Thai jasmine varieties stored for 12 months are characterised by an increase of particularly volatile compounds (Fig. 6d) as indicated by the strong positive loadings of PC2 (corresponding to the position of the jasmine samples stored for 12 months in Fig. 6c). In contrast, basmati samples are also characterised by an increase of semi-polar metabolites as analysed by DI–MS.

## Discussion

Basmati and jasmine rices are arguably the most popular rices in the world. Consumers pair these rices with appropriate cuisines, and can easily tell the difference between both types, on the basis of aroma, taste, flavour and texture (Champagne et al. 2010). However, breeding programs aiming to develop new varieties of each type are hampered by a lack of knowledge about ways to discriminate between the two rice types. Both types are fragrant, both express 2AP (Fitzgerald et al. 2008) as a major fragrance compound (Buttery et al. 1982), and both carry exactly the same genetic mutation that leads to the expression of 2AP (Kovach et al. 2009). Although factors such as grain appearance and cooked rice texture differ between jasmine and basmati rices selection for 2AP either by phenotype and/or genotype cannot discriminate the two classes. The analysis of volatile compounds in the headspace of aromatic rice has intensively been investigated over the years (Bryant and McClung 2011). However, all previous studies that included both jasmine and basmati varieties either used samples imported from Asia and grown in the country of study, or used commercial samples purchased from a local (super)market. Limitations to using these types of samples are that the pre- and post-harvest environments can have a significant effect on the volatile profile of jasmine and basmati rice (Yoshihashi et al. 2004)—as has also clearly been demonstrated here (Figs. 4, 6). Furthermore, basmati rice (Bhattacharjee et al. 2002), and commercial rice samples are often mixtures of several varieties, of uncertain origin (Sivaranjani et al. 2010; Steele et al. 2008).

The particular value of the collection of rice varieties used in the present study is that all were confirmed to be 100 % pure; grown specifically for this project, each in their adapted environment and conditions; and delivered with full harvest, postharvest and storage histories. Secondly, the samples have been analysed by six metabolomics platforms, each measuring mostly different groups of compounds. Results in Fig. 2 clearly demonstrate that the jasmine rices of the GMS and from other countries, all share common metabolic elements, which separates them at the first HCA cluster break, from the basmati samples. Deeper analysis of these data would provide the best opportunity yet to understand differences between basmati and jasmine rices to determine potential use for breeders, consumers and food chemists alike.

The results presented in Fig. 1a include all samples and data from all platforms. The most notable discrimination occurs on PC1 where the fresh basmati samples are shifted to the extreme of PC1. This is possibly explained by Fig. 1b, which shows that the polar and semi-polar compounds detected by NMR and DI-MS are generally polarized at the extremes of PC1. These compounds are mostly involved in primary metabolism of carbon and nitrogen (Moing et al. 2004). That the compounds from these three platforms separate fresh basmati samples from the others suggests the presence of specific metabolites or biochemical processes unique to fresh basmati and which appear to be no longer present after storage. Such changes should be borne in mind when assessing progeny for selection in any breeding programme. Consistent with this, Tables 2 and 3 reveal that, of the 30 most discriminating compounds from NMR and the DI–MS platforms, 14 are common in the comparisons between fresh and stored basmati (Table 2) and fresh basmati versus fresh jasmine (Table 3), signaling the importance of these compounds in discriminating fresh basmati samples from all the others. Basmati grains differ from all other types in that the grains only expand longitudinally when cooked (Bhattacharjee et al. 2002), which is attributed to the unique packing of starch granules and alignment of cells within the grains (Sekhar and Reddy 1982; Singh et al. 2000). It is therefore possible that the discriminating biochemical metabolites could be those participating in the pathways during grain-filling that lead to the unique internal arrangement of basmati grains. It is well known that next to the carbohydrate polymers, starch grains are highly complex and can comprise varying amounts of other components including lipids, sugars, amino acids, phosphorylated residues and mineral elements (Tester et al. 2004).

Jasmine rices are consumed as close to harvest as possible, while basmati consumers prefer to store their rice for about a year before consumption, an action which changes the flavour and aroma of the rice significantly (Bhattacharjee et al. 2002). Consumers can readily tell the difference between a jasmine and a basmati rice (Champagne et al. 2010), and Fig. 2 shows that the metabolomics analyses separate jasmine varieties from different parts of the world from the basmati varieties. In the present study, volatile and primary polar compounds in the grains of fresh jasmine and stored basmati rice are clearly different (Fig. 3a–c). However the compounds detected by DI-MS do not classify the types quite so well (Fig. 2d, e). None of the ten most discriminating volatile compounds, of which six were higher in basmati and four higher in jasmine, could be identified (Table 2). Seven of the ten most discriminating primary polar compounds were identified, but no clear biochemical relationship can be extrapolated. Nevertheless, the volatile and primary polar compounds are the most discriminating between these two types of rice, and are potentially valuable sources of markers, despite not yet being annotated.

Storage of basmati rice for 1 year leads to essential changes to the texture and cooking properties of the grains (Martin and Fitzgerald 2002), and also, the development of the typical basmati aroma and flavour (Bhattacharjee et al. 2002). All five platforms separated the fresh and stored basmati varieties (Fig. 4) indicating that the chemical changes occurring on storage are extensive. Again, only a few metabolites could be identified (Table 3), but we can speculate that lipid oxidation is likely to occur, leading to a different suite of odorous and malodorous compounds in the stored grains, such as fatty acids, aldehydes and furans (Zhou et al. 2003).

Even though we have demonstrated significant chemical differences between jasmine and basmati rices in the forms that they are generally used (i.e. fresh jasmine and stored/aged basmati) (Fig. 3; Table 2), as this stands, this is not useful to rice improvement programs, where selections are made on freshly harvested material. However, here we also demonstrate that freshly-harvested basmati and jasmine rice are clearly metabolically different (Fig. 5). All platforms could discriminate the types, providing potential opportunities for selection tools for breeding programs targeting both markets. While many of the discriminatory compounds have yet to be annotated, they can already be reliably detected by the platforms used, which is important for use in a breeding context. We have previously shown, in both rice and Arabidopsis, that metabolomic information from the platforms used can be exploited for association mapping, irrespective of any annotation (Calingacion et al. 2012; Fu et al. 2009; Keurentjes et al. 2006). Therefore, as long as compounds can be reliably detected, and heritability is confirmed, it should be possible in the future to identify genes that associate with these important discriminating, but as yet unidentified metabolites.

Stored basmati is not considered ‘rancid’ by basmati consumers whereas stored jasmine rice is (Laohakunjit and Kerdchoechuen 2007). Stored jasmine rice is often referred to as being ‘musty’ and ‘old’ and is unacceptable to consumers. On all platforms we see clearly that in jasmine rices metabolite profiles change as storage time increases (Fig. 6). Importantly, the metabolic shifts do not involve the same compounds that discriminate between fresh and stored basmati rice. This indicates that aging proceeds differently in the two types of rice, in agreement with consumer data. Interestingly, it is clear that RD 33, RD 6 and KDML 105 differ from PTT 1 when freshly harvested, but after 12 months of storage at room temperature, there appears to be little difference between the metabolomes of KDML and PTT 1 (Fig. 6). KDML 105 is marketed in the high quality Hom Mali class but although PTT 1 is not (Pitiphunpong et al. 2011), it is often found as an adulterant in rice marketed as Hom Mali rice (Attaviroj et al. 2011). The data presented here can (i) be used to support policy development in Thailand in the areas of rice marketing, storage, and stockpiling, and (ii) provide potential tools for detecting adulteration of the Hom Mali class in fresh samples of rice.

The jasmine variety PTT 1, which has been improved through breeding for a number of agronomic characteristics, does not cluster with the other Thai jasmine rices (Figs. 2, 6), and indeed, was often misclassified in the other comparisons. This variety is a semi-dwarf jasmine rice that has undergone several breeding cycles (www.irri.org). RD 6 was a product of mutation breeding using KDML 105 (Somrith 1998), and RD 33 is the product of a back-crossing program using a variety carrying blast resistance as the donor parent and KDML105 as the recurrent parent (Chamarek et al. 2008). Therefore RD 33 and RD 6 are both derivatives of KDML 105 while PTT 1 is not. Recently, the genetic structure of Thai rices was determined, and PTT 1, while falling close to the other Thai rices, was one of two genetically isolated accessions (Chakhonkaen et al. 2012). This is consistent with the biochemical compositional differences between PTT 1 and the other jasmine rices in many of the datasets as observed here.

As with many other crops in the past, breeding for rice quality has often taken second place to crop yield and disease resistance. This was primarily because of a preoccupation with productivity at the start of the green revolution, and subsequent to that, limited resources and the lack of a sufficient number of appropriate tools for quality evaluation as a means to assist the rice breeder to select also for quality attributes (Calingacion et al. 2014). Large-scale sensory analyses are limited because of the high costs and the often small amount of material that is produced during cultivar development. Systematic robust, non-subjective, predictive models for quality evaluation are therefore needed to allow us to visualise and exploit key traits and subsequently, help support direct breeding strategies (Limpawattana et al. 2008). For this we need a broad-based analytical approach to complement already-existing genome-based approaches (e.g. Zhao et al. 2010). Metabolomics platforms have the potential to bridge this gap in our analytical potential, and in combination with other methodologies such as Genotyping by Sequencing (GBS), we have developed a powerful approach not only to understand the biochemical differences but also the genetics driving these. Their further use in helping us to continue to build a more detailed picture of the complexity of rice aroma and rice taste will be a very welcome addition to the plant breeders arsenal of tools required for breeding new high-yielding *and* high-quality rice varieties and targeting these accurately to specific local and international export markets. A logical next step would be to link the biochemistry determined by genetics as described here (which plays a major role in consumer choice in a retail situation) to the biochemistry resulting from cooking in order to understand better how the natural biochemical profile is converted into the processed one which plays the second major role by determining consumer preference at kitchen level.

## Conclusions

Here, we demonstrate what rice consumers of course already know, that grains from jasmine and basmati rice have significantly different metabolomes which is consistent with recognized differences in sensory properties. We can now specify these differential properties and the most significant differences were found in the groups of volatile (SPME–GC–MS) and primary polar compounds (GC–TOF–MS) suggesting that these platforms have greatest potential value in future application. Importantly, we have also shown that the differences in biochemical profile between the two types is not simply a function of ageing. With all platforms, fresh Thai jasmine rice and fresh basmati rice were clearly found to differ, thus providing the potential to develop selection tools for breeding programs. Furthermore, we have shown differences between major Thai rice varieties that can lead to policy development in important areas of rice marketing. The next steps are to focus on annotating the discriminating compounds and to develop a conduit of delivery to rice breeding programs to enable robust selection of traits that define sensory and cooking quality.

## Electronic supplementary material

Supplementary material 1 (XLSX 11 kb)
